# Work Engagement as a Predictor of Onset of Major Depressive Episode (MDE) among Workers, Independent of Psychological Distress: A 3-Year Prospective Cohort Study

**DOI:** 10.1371/journal.pone.0148157

**Published:** 2016-02-03

**Authors:** Kotaro Imamura, Norito Kawakami, Akiomi Inoue, Akihito Shimazu, Akizumi Tsutsumi, Masaya Takahashi, Takafumi Totsuzaki

**Affiliations:** 1 Department of Mental Health, Graduate School of Medicine, The University of Tokyo, Tokyo, Japan; 2 Department of Mental Health, Institute of Industrial Ecological Sciences, University of Occupational and Environmental Health, Japan, Kitakyushu, Japan; 3 Department of Public Health, Kitasato University School of Medicine, Sagamihara, Japan; 4 Health Administration and Psychosocial Factor Research Group, National Institute of Occupational Safety and Health, Japan, Kawasaki, Japan; 5 Uchisaiwaicho Medical Center, Mizuho Health Insurance Society, Tokyo, Japan; National Center of Neurology and Psychiatry, JAPAN

## Abstract

**Objective:**

This study investigated work engagement as a baseline predictor of onset of major depressive episode (MDE).

**Methods:**

The study used a prospective cohort design, conforming to the STROBE checklist. Participants were recruited from the employee population of a private think tank company (N = 4,270), and 1,058 (24.8%) of them completed a baseline survey, of whom 929 were included in this study. Work engagement and psychological distress at baseline were assessed as predictor variables. MDE was measured at baseline and at each of the follow-ups as the outcome, using the web-based, self-administered version of the Japanese WHO-CIDI 3.0 depression section based upon DSM-IV-TR/DSM-5 criteria. Cox discrete-time hazards analyses were conducted to estimate hazard ratios (95% confidence intervals CIs).

**Results:**

Follow-up rates of participants (N = 929) were 78.4%, 67.2%, and 51.6% at 1-, 2-, and 3-year follow-ups, respectively. The association between work engagement at baseline and the onset of MDE was U-shaped. Compared with a group with low work engagement scores, groups with the middle and high scores showed significantly (HR = 0.19, 95% CI = 0.05 to 0.64; p = 0.007) and marginally significantly (HR = 0.48, 95% CI = 0.20 to 1.15, p = 0.099) lower risks of MDE, respectively, over the follow-ups, after adjusting for covariates. The pattern remained the same after additionally adjusting for psychological distress.

**Conclusions:**

The present study first demonstrated work engagement as an important predictor of the onset of MDE diagnosed according to an internationally standard diagnostic criteria of mental disorders.

## Introduction

Depressive disorder is one of the most prevalent psychiatric disorders and is associated with a substantial deterioration in quality of life and economic loss in the community and workplace [1, 2]. Identifying work related predictors of depressive disorder is an important strategy for preventing the disorder and promoting mental health and well-being among workers.

Recently, research in occupational health has focused on positive mental health outcomes [3]. One such outcome is work engagement, which is a positive, fulfilling, work-related state of mind and measured with three dimensions: vigor, dedication, and absorption [4, 5]. A previous longitudinal study showed that work engagement was positively associated with quality of life of employees (i.e., job and family satisfaction) and productivity [6, 7]. On the other hand, the association of work engagement with mortality and morbidity was less clear. A previous cross-sectional survey reported that work engagement did not have any significant relationship with the treatment for health conditions (e.g., cardiovascular condition, high cholesterol, depression, diabetes, hypertension and irritable bowel syndrome) [8]. However, another one-year cohort study reported an L-shaped association between work engagement and high-sensitivity C-reactive protein (hs-CRP) levels, a risk factor for cardiovascular diseases [9]. Participants with moderate and high levels of work engagement at baseline had significantly lower odds ratios (ORs) of having high hs-CRP levels at follow-up than those with low levels of work engagement at baseline. However, having high levels of work engagement had no additional benefits compared to having moderate levels of work engagement.

No previous study investigated work engagement as a predictor for the onset of depressive disorder. For non-clinical mental health outcomes, two previous studies showed that work engagement at baseline was negatively associated with non-clinical depression and anxiety at follow-up [10, 11]. Beside work engagement, a previous study reported that satisfaction with oneself predicted a lower risk of major depressive episode (MDE) during a follow-up among women [12]. These general positive emotions could be theoretically different from work engagement in many aspects, in that work engagement is affect and attitude more related to and specific to work settings [6]. However, these pieces of evidence suggest that (low) work engagement is a predictor of the future onset of MDE.

Work engagement is negatively associated with psychological distress (e.g., non-clinical depression and anxiety) cross-sectionally [13]. At the same time, psychological distress is known as a strong predictor of the onset of MDE [14]. Thus, even if work engagement is found as a predictor of MDE, the effect of work engagement may be a shadow of that of psychological distress. Psychological distress could be a confounder or mediator to be considered in investigating the association between work engagement and the future onset of MDE. It would be interesting to know whether work engagement predicts MDE independent of psychological distress.

The aim of this study was to investigate whether baseline work engagement could predict the onset of major depressive episode (MDE) diagnosed according to DSM-IV/DSM-5 [15, 16] during a 3-year follow-up. Psychological distress was adjusted for as a covariate to know whether work engagement predicts the onset of MDE independently of psychological distress.

## Materials and Methods

### Study Design

The present study was a prospective cohort designed as a part of the occupational cohort study on social class and health conducted in Japan (Japanese Study of Health, Occupation, and Psychosocial Factors Related Equity: J-HOPE). The present analysis was conducted with the J-HOPE dataset as of 22 August 2014. The Research Ethics Review Board of the University of Tokyo, Graduate School of Medicine (No. 2772) approved the study procedures. Study purposes and procedures were explained and written informed consent was obtained from the employees prior to the initiation of the study. The present study conformed to the STROBE checklist.

### Participant Recruitments

The participants of the present study were recruited by means of an invitation e-mail from their company management from a private think tank company (N = 4,270). All outcome, predictor, and potential confounder variables were measured using a web-based self-report questionnaire at baseline and annual 3-year follow-up survey. The baseline survey was conducted between October 2010 and March 2011. The follow-up surveys were conducted at three times on an annual basis. The first year follow-up survey was conducted between December 2011 and January 2012. The second year follow-up survey was conducted between December 2012 and January 2013. The third year follow-up survey was conducted between January 2014 and February 2014. The inclusion criteria at the baseline survey were (1) currently employed full-time by the business company and (2) a Japanese ability to understand the scope of the study and to provide written consent for study participation. The exclusion criteria were (1) having a major depressive disorder in the past year (using diagnostic criteria on the web version of the WHO-CIDI 3.0 [17, 18] and (2) receiving medical treatment for mental health problems during the past month.

### Outcome Variable

#### Dependent variable: incidence of MDE

The outcome was the onset of MDE during the annual three-year follow-ups. The onset of MDE during the follow-up was assessed using the web-based self-administered version of the Japanese WHO-CIDI 3.0 depression section [19, 20], according to DSM-IV-TR criteria [15, 16]. We did not exclude bereavement from the diagnosis of MDE; the diagnostic criteria for this study were identical to that of the most recent version of DSM-5 [15, 16]. Only MDE that occurred during the previous 12 months was assessed for this study. The web-version has been shown to have a good concordance with the clinical diagnosis of MDE [21] and be reliable in a one-year test-retest survey [18].

#### Predictor variables: Work engagement

Work engagement was assessed using the short form of the Japanese version of the Utrecht Work Engagement Scale (UWES) [22]. The UWES consists of 3 subscales comprising 9 items (e.g., vigor, dedication, absorption). Items are scored on a 7-point scale ranging from 0 (never) to 6 (always). Item examples are “At my job, I feel strong and vigorous” (vigor), “I am enthusiastic about my job” (dedication), and “I am immersed in my work” (absorption). A total score was calculated from all 9 items, and then the total score was averaged to get an average score.

#### Predictor variables: Psychological distress

Psychological distress was measured by the Japanese version of Kessler’s Psychological Distress Scale (K6) [23, 24]. The K6 scale consists of six items assessing the frequency with which respondents have experienced symptoms of psychological distress during the past 30 days. The response options range from 0 (none of the time) to 4 (all of the time). The internal reliability and validity found in previous studies are acceptable [23].

#### Potential confounder: demographic characteristics

Demographic characteristics included sex (male or female), age (20–34, 35–44, or 45+), education (“high school or some college” or “university or higher”), occupation (manager, professionals, or technician/clerk/others), household income (“less than 8” or “8 or more” million yen per year), living with family (no or yes), daily drinking (“none or occasionally”, or “daily”), and chronic conditions (none or any) were collected as the covariates.

### Sample Size Calculation

The post-hoc sample size calculation was conducted in the present study. There is no appropriate previous study to estimate the incident ratio (IR) in this study. A previous follow-up survey of employees in a company showed that the incidence of major depressive disorder was 2.8% during twelve months [18]. We applied a method proposed by Rubinstein and colleagues [25] to calculate a minimal sample size and a statistical power for a proportional hazard model analysis. Thus, with a population of 4,326 in this study, we have 75% power to detect a predictive value, assuming that IR = 0.5. If the participation rate was 25% and we had only about 1,000 participants, the statistical power would be 25%. These calculations ignore dropout. If only 70% of these 1,000 participants complete the 3-year follow-up, the power would be 19%.

### Statistical Analysis

The predictive association between work engagement and incident MDE was investigated over the study period with semiparametric discrete-time Cox proportional hazards models, to estimate hazard ratio (HR), with 95% confidence intervals (CIs), while controlling for censoring effects due to the differential length of follow-up or the completion of follow-up without the onset of MDE. The predictive variables (work engagement and psychological distress) were used as the categorical variables, which were divided into tertiles (high, middle, and low). We applied two models to adjust for covariates. Model 1 was adjusted for sex and age. In addition to model 1, model 2 was adjusted for all potential confounders: categorical variables of education, occupation, household income, living with family, daily drinking, and chronic conditions. To examine the predictive association between work engagement and incident MDE, independent of psychological distress, a series of analyses were conducted. In the first step, each of work engagement and psychological distress was used as the predictive factor in a separate analysis. In the next step, psychological distress was used as the covariate to test whether work engagement predicts the onset of MDE independently. The ability of work engagement and psychological distress to predict MDE was also examined by time-dependent receiver operating characteristic (ROC) curve analysis [26]. Analyses were done with SPSS version 21 and R software programming (http://www.r-project.org). A significance level of less than 0.05 was used and all tests were two-tailed.

## Results

### Participant Flowchart

**Fig 1:**
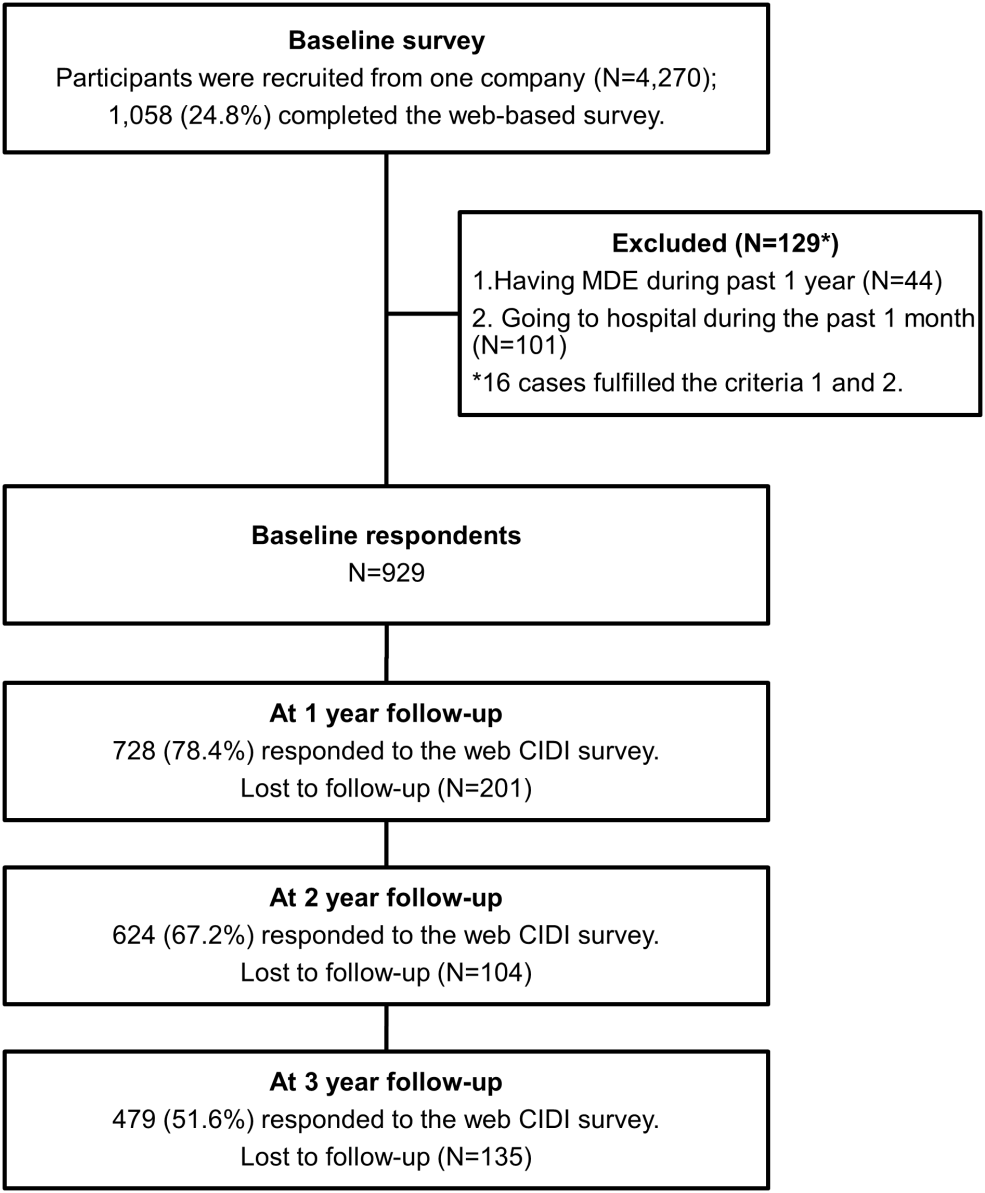
Participant flow diagram.

Fig 1 shows the Participant flowchart in this study. Participants were recruited from one company (N = 4,270), and 1,058 (24.8%) of them completed a baseline survey. Out of those, 129 had to be excluded because 44 fulfilled the exclusion criterion #1 (diagnosed as major depressive disorder in the past 1 year, which were assessed using the web-version of WHO-CIDI 3.0), and 101 fulfilled the exclusion criterion #2 (having gone to the hospital during the past 1 month). Sixteen of them fulfilled both criteria #1 and #2. The remaining 929 participants were included in this study. At 1-year follow-up, 728 (78.4%) participants completed the follow-up survey. At the 2-year follow-up, 624 (67.2%) participants completed the follow-up survey. At the 3-year follow-up, 479 (51.6%) participants completed the follow-up survey. Reasons for dropping out were not assessed in this study.

### Baseline Characteristics

Table 1 shows the demographic characteristics at baseline survey. Average age (standard deviations) was 38.4 (8.7). Most participants were males (77.6%), graduates of university or higher education (82.9%), and living with family (67.8%). Half of the participants were employed as professionals (50.4%); the others worked as managers or technician/clerk/others. A small proportion of the participants had daily alcohol consumption habit and chronic disease (25.2% and 17.9%, respectively).

**Table 1:** Characteristics of 929 respondents employed for an information systems developing company in Japan who did not have major depressive episode or receive mental health care in the past year.

	n	%	Average	SD
Sex				
Male	721	77.6%		
Female	208	22.4%		
Age group (years)			38.4	8.7
20–34	333	35.8%		
35–44	332	35.7%		
45+	264	28.4%		
Occupation				
Manager	259	27.9%		
Professionals	468	50.4%		
Technician/clerk/others	202	21.7%		
Educational attainment				
High school or some college	208	22.4%		
University or higher	770	82.9%		
Household income (million yen per year)				
Less than 8	436	46.9%		
8 or more	493	53.1%		
Living with family				
No	299	32.2%		
Yes	630	67.8%		
Daily drinking				
None or occasionally	695	74.8%		
Daily	234	25.2%		
Chronic conditions				
None	763	82.1%		
Any	166	17.9%		
Psychological distress (K6)			5.2	4.5
Work engagement (UWES)			2.9	0.9

### Incidence of MDE associated with Work Engagement and Psychological Distress

Among 1,807 person-years observation during the 3-year follow-up, a total of 27 participants reported a new onset of MDE, with an incidence of 1.5% per year. Table 2 shows the incidence rates for MDE among the groups classified on the basis of work engagement or psychological distress at baseline, along with sex and age-adjusted HRs and fully-adjusted HRs. Among the three groups divided according to work engagement score at baseline, the incidence rate of MDE was lowest in the middle-score group and highest among low-score group. Compared with the low-score group, the middle-score group showed a significantly lower HR after adjusting for sex and age (HR 0.18, 95% confidence interval [CI] 0.05 to 0.62, p = 0.007) and after being fully-adjusted (HR 0.19, 95% CI 0.05 to 0.64, p = 0.007). Among the three groups divided according to psychological distress score at baseline, the incidence of MDE was highest in the high-score group, followed by the middle score group. Compared with the low-score group, the high-score group showed a significantly higher HR after adjusting for sex and age (4.24, 95% CI 1.40 to 12.86, p = 0.011) and after being fully-adjusted (4.02, 95% CI 1.30 to 12.37, p = 0.015).

**Table 2:** Relative risks (hazard ratios, HRs)^†^ of major depressive episode (MDE) during three-year follow-up associated with work engagement or psychological distress at baseline among 929 employees of an information systems developing company in Japan who did not have MDE in the past year or never received mental health care.

					Sex and age-adjusted			Fully-adjusted^‡^		
Variable (score ranges)	n	Total person-years observed	New MDE cases (n)	Incidence (/year)	HR	95%CI	p	HR	95%CI	p
Work engagement (UWES)										
Low (0–2.59)	314	566	16	0.028	1			1		
Middle (2.60–3.19)	286	588	3	0.005	0.18	0.05 to 0.62	0.007	0.19	0.05 to 0.64	0.007
High (3.20+)	329	653	8	0.012	0.45	0.19 to 1.06	0.069	0.48	0.20 to 1.15	0.099
					(p = 0.012, df = 2)			(p = 0.016, df = 2)		
Psychological distress (K6)										
Low (0–2)	325	687	4	0.006	1			1		
Middle (3–6)	300	578	7	0.012	2.00	0.58 to 6.84	0.269	1.96	0.57 to 6.74	0.285
High (7+)	304	542	16	0.030	4.24	1.40 to 12.86	0.011	4.02	1.30 to 12.37	0.015
					(p = 0.023, df = 2)			(p = 0.035, df = 2)		

^†^ Cox's discrete proportional hazard model.

^‡^ Adjusted for sex, age groups, occupation, education, household income, living with family, daily drinking, and chronic condition.

Table 3 shows the associations (HRs) of work engagement and psychological distress with MDE, simultaneously adjusting for these two predictors and all covariates in a model. Compared with the group with low scores of work engagement, the middle-score group still showed a significantly lower HR (0.21, 95% CI 0.06 to 0.74, p = 0.015). Compared with the group with low scores of psychological distress, the high score group showed a significantly higher HR (3.41, 95% CI 1.08 to 10.77, p = 0.036).

**Table 3:** Relative risks (hazard ratios, HRs) of major depressive episode (MDE) during three-year follow-up associated with work engagement and psychological distress at baseline among 929 employees of an information systems developing company in Japan who did not have MDE in the past year or never received mental health care^†^.

Variables at baseline	HR	95%CI		p
Work engagement (UWES)				
Low (0–2.59)	1			
Middle (2.60–3.19)	0.21	0.06 to 0.74		0.015
High (3.20+)	0.60	0.25 to 1.45		0.257
	(p = 0.045, df = 2)^‡^			
Psychological distress (K6)				
Low (0–2)	1			
Middle (3–6)	1.95	0.56 to 6.76		0.291
High (7+)	3.41	1.08 to 10.77		0.036
	(p = 0.092, df = 2)^‡^			
Sex				
Men	1			
Women	1.23	0.51 to 2.97		0.653
Age group (years)				
22–44	1			
45–54	0.78	0.32 to 1.94		0.597
55–63	0.31	0.07 to 1.34		0.118
	(p = 0.291, df = 2)^‡^			
Occupation				
Manager	1			
Professionals	0.87	0.20 to 3.77		0.853
Technician/clerk/others	1.07	0.23 to 5.04		0.933
	(p = 0.897, df = 2)^‡^			
Educational attainment				
High school or some college	1			
University or higher	0.59	0.23 to 1.54		0.279
Household income (million yen per year)				
Less than 8	1			
8 or more	0.92	0.30 to 2.84		0.883
Living with family				
No	1			
Yes	0.71	0.28 to 1.78		0.461
Drinking				
None or occasionally	1			
Daily	0.46	0.13 to 1.57		0.215
Chronic conditions				
None	1			
Any	1.54	0.54 to 4.42		0.421

^†^ Cox's discrete proportional hazard model. All variables were simultaneously entered in the model.

^‡^ Test for significant difference across three categories.

Table 4 shows results of similar discrete-time Cox proportional hazards model analyses (models 1 and 2) for each subscale (i.e., vigor, dedication, or absorption) of work engagement. The association between vigor or absorption and MDE was somewhat U-shaped, while that for dedication was linear. Compared with the group with low scores of vigor, the middle-score group showed a significantly lower HR (0.37, 95% CI 0.14 to 0.98, p = 0.045). Similarly, compared with the group with low scores of absorption, the middle-score group showed a significantly lower HR (0.33, 95% CI 0.12 to 0.91, p = 0.033). However, compared with the group with low scores of dedication, the high-score group showed a lower HR, while it was not significant.

**Table 4:** Relative risks (hazard ratios, HRs)^†^ of major depressive episode (MDE) during three-year follow-up associated with the subscales of work engagement at baseline among 929 employees of an information systems developing company in Japan who did not have MDE in the past year or never received mental health care.

					Sex and age-adjusted			Fully-adjusted^‡^		
Variable (score ranges)	n	Total person-years observed	New MDE cases (n)	Incidence (/year)	HR	95%CI	p	HR	95%CI	p
Vigor										
Low (0–2.34)	290	522	14	0.027	1			1		
Middle (2.66–3.00)	349	707	6	0.008	0.31	0.12 to 0.80	0.016	0.37	0.14 to 0.98	0.045
High (3.33+)	290	578	7	0.012	0.45	0.18 to 1.11	0.082	0.63	0.24 to 1.65	0.350
					(p = 0.032, df = 2)			(p = 0.128, df = 2)		
Dedication										
Low (0–2.67)	356	653	15	0.023	1			1		
Middle (3–3.34)	307	619	8	0.013	0.58	0.24 to 1.38	0.220	0.69	0.29 to 1.65	0.405
High (3.66+)	266	535	4	0.007	0.35	0.12 to 1.08	0.067	0.47	0.15 to 1.49	0.201
					(p = 0.142, df = 2)			(p = 0.395, df = 2)		
Absorption										
Low (0–2.34)	324	607	15	0.025	1			1		
Middle (2.66–3.00)	303	620	5	0.008	0.33	0.12 to 0.90	0.030	0.33	0.12 to 0.91	0.033
High (3.33+)	302	580	7	0.012	0.48	0.19 to 1.18	0.108	0.54	0.22 to 1.37	0.195
					(p = 0.056, df = 2)			(p = 0.080, df = 2)		

^†^ Cox's discrete proportional hazard model.

^‡^ Adjusted for sex, age groups, occupation, education, household income, living with family, daily drinking, chronic condition, and K6.

### Predictive Performance of Work Engagement and Psychological Distress for the Onset of MDE

**Fig 2:**
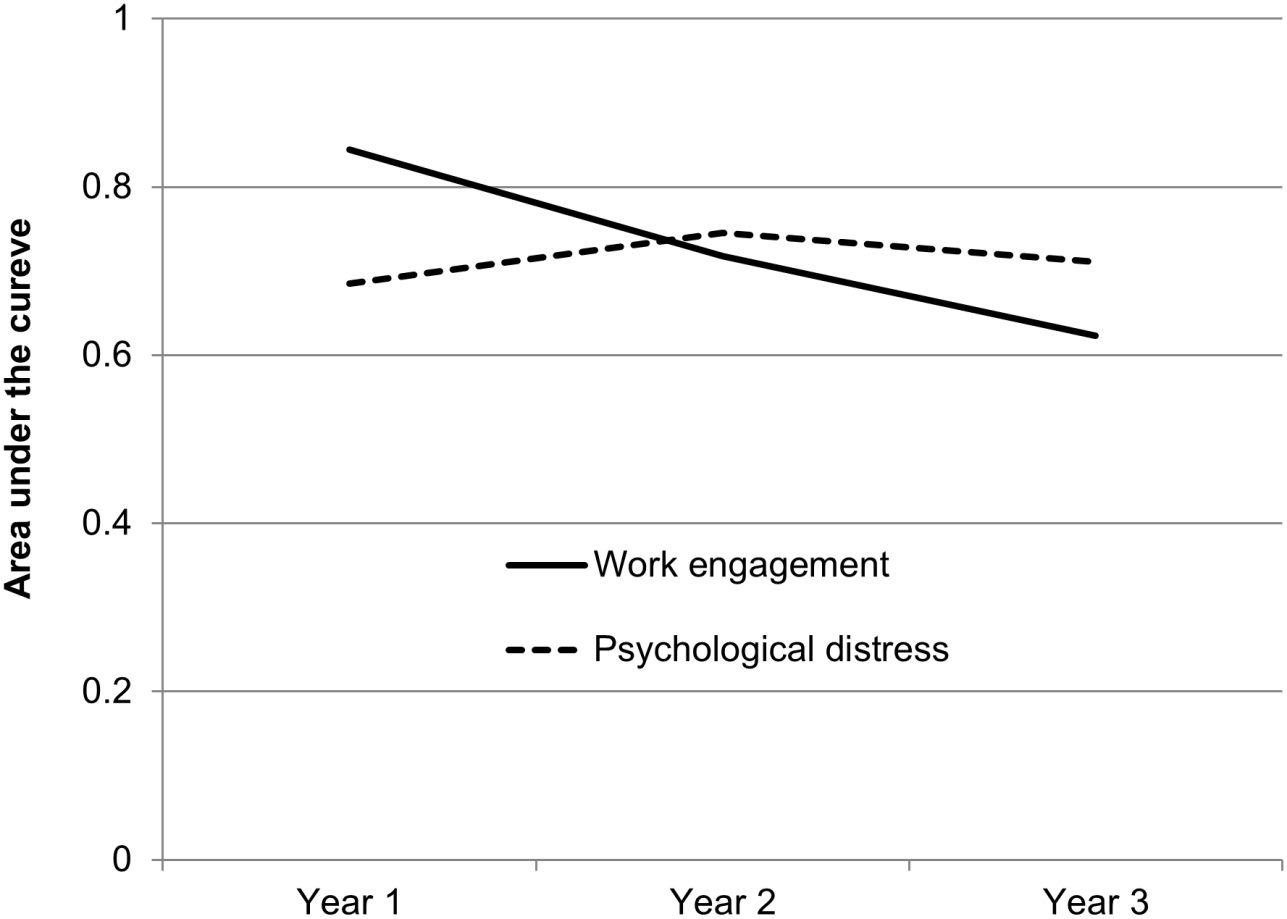
Predictive performance (area under the curve, AUC) of work engagement (UWES) and psychological distress (K6) for the onset of major depressive episode by the follow-up years: Estimated by survival Receiver Operating Characteristics (ROC) curve.

Time-dependent ROC curves over the entire follow-up period are presented in Fig 2. For work engagement, AUC (area under the curve) values for MDE were 0.844, 0.718, and 0.623 at 1-, 2-, and 3-year, respectively. For psychological distress, AUC values for MDE were 0.685, 0.745, and 0.711 at 1-, 2-, and 3-year, respectively.

## Discussion

The present prospective cohort study examined the predictive performance of work engagement for the onset of MDE among workers at 3-year follow-up. In the results, the group with low scores of work engagement at baseline had a significantly higher risk for the onset of MDE than the groups with middle scores. The association between work engagement and MDE was U-shaped, with higher risks of MDE among both groups with high and low scores. The association between work engagement and MDE was independent of psychological distress.

In the present study, the group with low work engagement had the highest risk for MDE among the three groups classified on the basis of work engagement at baseline. This result is consistent with previous studies which reported that work engagement predicted non-clinical depression and anxiety [10, 11]. It is also consistent with a previous observation that positive emotion (i.e. satisfaction with oneself) predicted a lower risk of MDE [12]. The present study first demonstrated that work engagement may be an important predictor of the onset of MDE diagnosed according to the internationally standard diagnostic criteria of mental disorders, DSM-IV/DSM-5. Interestingly, the association of work engagement with the onset of MDE remained the same after adjusting for psychological distress. Work engagement may have its own predictive value for the onset of MDE, not just reflecting a lack of psychological distress.

The present study showed a U-shaped association between work engagement and MDE. The onset of MDE was more frequent in the group with high work, as well as low, engagement than that with middle level of work engagement. A similar U-shaped association was reported by a previous 1-year prospective cohort study which showed that hs-CRP levels were high both among groups with low and high work engagement [9]. High levels of work engagement may not be health-promoting, or even have an adverse effect on health, because work engagement may be followed by greater workload or overtime [27], work engagement may cause high arousal which increases reactivity in the hypothalamic, pituitary, adrenal (HPA) axis [28], or high work engagement may be contaminated by workaholism, a maladaptive work-related behavior which is associated with poor health status [13]. Workers with high work engagement may not enjoy a full merit from having positive emotions/attitude to work because of any of these reasons. Further research should investigate further the mechanisms with which workers with high and low work engagement have compromised health.

Among the three subscales of UWES, a similar U-shaped association was observed for vigor and absorption. On the other hand, dedication showed an almost linear association with MDE. Vigor is characterized by high levels of activation, energy and mental resilience while working [4]. Absorption is characterized by being fully concentrated and deeply engrossed in one’s work [4]. These components of work engagement could be associated with higher arousal level of workers. It is known that hyperactivity of the HPA-axis is a part of neuro-endocrinological pathology of MDE [29, 30]. High levels of vigor and absorption, which reflect high levels of arousal, may increase a future risk of MDE. On the other hand, dedication is characterized by a sense of significance, enthusiasm, inspiration, pride, and challenge. Positive judgments about the meaning and purpose of working life may be associated with psychological resources, such as self-esteem and sense of control, which are known as a protective factor for MDE [31]. This may be a reason that dedication was linearly associated with MDE. Although work engagement was treated as a unitary construct due to high correlations among three components [22], future study needs to treat these components as independent constructs because they have different functions to future health outcomes. The present findings should be replicated with a larger sample with further investigation of psychological and physiological mediators, possibly linking the components of work engagement to MDE.

In the present study, the group with high and moderate psychological distress had 4.02 and 1.96 times higher the risk of MDE, respectively, (when fully adjusted for the covariates) compared with the group with low psychological distress. A population attributable risk percent (PARP) was estimated as 0.565 in this sample. The result is consistent with previous findings that negative emotions were a strong predictor of MDE [10, 11]. However, in this study, the groups with low work engagement showed about 5 times greater risk of MDE compared to the group with moderate work engagement. The PARP calculated for work engagement was 0.682. Work engagement should be recognized to be as important a predictor of MDE as psychological distress has been. Workplace interventions to improve work engagement at least among workers with low work engagement might be an alternative strategy to prevent MDE at workplace. In the present study, for the group of moderate levels of work engagement, UWES scores ranged from 2.60 to 3.19, which may be useful identify groups with low and high work engagement in terms of a risk of MDE in Japan. However, an optimal cut-off score of UWES to predict MDE should be carefully investigated with a larger sample. In addition, average scores of UWES vary across countries and cultures [32]. Such an optimal cut-off score should be tested in other countries.

Time-dependent AUC for WE to predict MDE was moderate, but comparable to that for psychological distress. However, the AUC for work engagement was largest at the first year follow-up, then the AUC decreased gradually in the second and third year. On the other hand, the AUC remained stable over time for K6. Work engagement may be more accurate, thus useful to predict the onset of MDE within a shorter period (e.g., within one-year), while psychological distress may predict the onset of MDE for a longer time. Such a different time-dependent predictor power may depend on the stability over time, the mechanism to be associated with MDE, and the latent period from an exposure to the onset of each indicator. For instance, work engagement may be a state marker of a brain function which is protectve against MDE, while psychological distress could be a marker of the core psychopathology of depressive disorder. Future research is warranted to investigate the differential nature of predicting MDE between work engagement and psychological distress.

### Limitations

Possible limitations of the present study should be considered. One of the major limitations is that MDE was not diagnosed by a clinician, but measured by self-report, which might have been affected by the perception of participants or by situational factors at work. In addition, the validity of the web-based CIDI depression section still needs further validation, because the CIDI can measure the episodes more strictly following DSM-IV criteria. Second, participants were recruited from one private think tank company in Japan. Most participants were males, professionals, and university graduates. The generalization of the present findings to the entire working population is thus somewhat limited. While we did not assess differences in demographic and other characteristics between the target population and the study sample, it was well expected that the study sample may be either more depressed or more engaged to be interested in participating in the study. This might shift the classification of the tertiles of psychological distress and/or work engagement to the extremes, resulting in underestimation of the associations. Third, the dropout rates from the follow-up surveys in this study were 21.6%, 32.8%, and 48.4% at 1-, 2- and 3-year follow-ups, respectively. Dropouts may have caused a loss to follow-up bias, particularly if respondents in each follow-up survey had higher levels of interest in this survey and were healthier. In addition, the dropout rate in the present study was larger than that of the estimation in the post-hoc sample size calculation in the present study. A statistical power would be assumed to be even lower than 19%. Further replication study would be needed under appropriate sample size to detect the difference. Fourth, the initial response rate was low (24.8%). Candidates who were willing to participate in the present study may have responded to the baseline survey. This may limit the generalization of the present findings, while the mean score of UWES at baseline were same as the previous study [27]. Fifth, all outcomes in this study were measured by self-report, which may be affected by participants’ perceptions or situational factors at work. A self-reported measure could be vulnerable to a cognitive bias. A further prospective cohort study should be conducted to examine whether work engagement predicts the onset of MDE in a representative sample of workers with diverse characteristics, particularly in terms of occupation and education.

## Supporting Information

S1 File STROBE checklist of information to include when reporting a cohort studies. (DOC)
