# Correction: Work Engagement as a Predictor of Onset of Major Depressive Episode (MDE) among Workers, Independent of Psychological Distress: A 3-Year Prospective Cohort Study

**DOI:** 10.1371/journal.pone.0167862

**Published:** 2016-12-02

**Authors:** Kotaro Imamura, Norito Kawakami, Akiomi Inoue, Akihito Shimazu, Akizumi Tsutsumi, Masaya Takahashi, Takafumi Totsuzaki

There are multiple errors in the body of the manuscript and Fig 1.

The last sentence of the ‘Participant Recruitment’ section in the Methods is incorrect. The correct sentence should be: The exclusion criteria were (1) having a major depressive disorder in the past year (using diagnostic criteria on the web version of the WHO-CIDI 3.0 [17, 18] and (2) ever had a diagnosis of or received treatment for mental disorder.

The second and third to last sentences of the ‘Participant Flowchart’ section of the Results have incorrect percentages. The correct sentences are: At the 2-year follow-up, 644 (69.3%) participants completed the follow-up survey. At the 3-year follow-up, 496 (53.3%) participants completed the follow-up survey.

There is an error in the ‘At 2 year follow-up’ and ‘At 3 year follow-up’ percentages presented in Fig 1. Please view the correct Fig 1 here.

**Fig 1:**
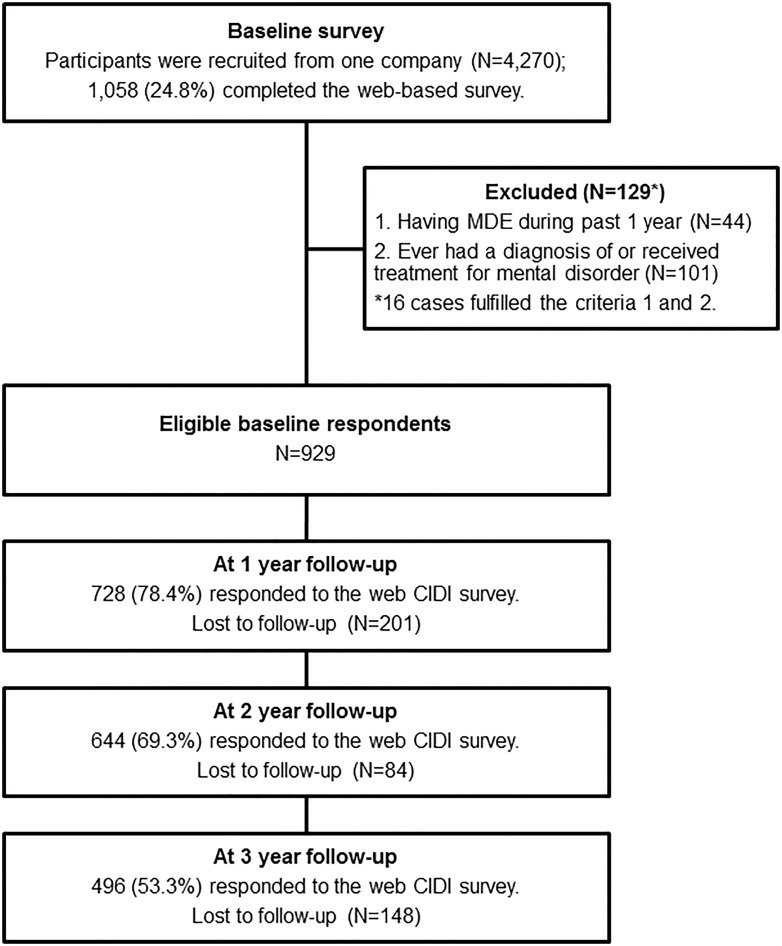
Participant flow diagram.
